# Author Correction: Mechanical performance of sustainable high strength ductile fiber reinforced concrete (HSDFRC) with wooden ash

**DOI:** 10.1038/s41598-022-09020-3

**Published:** 2022-03-18

**Authors:** Jawad Ahmad, Rebeca Martínez‑García, Jesús de‑Prado‑Gil, Amjad Ali Pasha, Kashif Irshad, Mostefa Bourchak

**Affiliations:** 1 Department of Civil Engineering, Swedish College of Engineering and Technology, Wah Cantt, Rawalpindi, Pakistan; 2 Department of Mining Technology, Topography, and Structures, University of León, Campus de Vegazana s/n, 24071 León, Spain; 3 Aerospace Engineering Department, King Abdulaziz University, Jeddah, 21589 Saudi Arabia; 4 Interdisciplinary Research Center for Renewable Energy and Power Systems, King Fahd University of Petroleum and Minerals, Dhahran, 31261 Saudi Arabia; 5 K.A.CARE Energy Research & Innovation Center at Dhahran, Dhahran, Saudi Arabia

Correction to: *Scientific Reports* https://doi.org/10.1038/s41598-022-08134-y, published online 12 March 2022

The original version of this Article contained an error in Affiliation 5, which was incorrectly given as ‘K.A.CARE Energy Research & Innovation Center, King Fahd University of Petroleum and Mineral, Dhahran, Saudi Arabia’. The correct affiliation is listed below.

K.A.CARE Energy Research & Innovation Center at Dhahran, Saudi Arabia.

The original Article has been corrected.

